# Quantification of DNA Double Strand Breaks and Oxidation Response in Children and Adults Undergoing Dental CBCT Scan

**DOI:** 10.1038/s41598-020-58746-5

**Published:** 2020-02-07

**Authors:** Niels Belmans, Liese Gilles, Randy Vermeesen, Piroska Virag, Mihaela Hedesiu, Benjamin Salmon, Sarah Baatout, Stéphane Lucas, Ivo Lambrichts, Reinhilde Jacobs, Marjan Moreels, DIMITRA Research Group, A. C. Oenning, C. Chaussain, C. Lefevre, M. Baciut, M. Marcu, O. Almasan, R. Roman, I. Barbur, C. Dinu, H. Rotaru, L. Hurubeanu, V. Istouan, O. Lucaciu, D. Leucuta, B. Crisan, L. Bogdan, C. Candea, S. Bran, G. Baciut, H. Bosmans, R. Bogaerts, C. Politis, A. Stratis, R. Pauwels, K. de F. Vasconcelos, L. Nicolielo, G. Zhang, E. Tijskens, M. Vranckx, A. Ockerman, E. Claerhout, E. Embrechts

**Affiliations:** 1 Morphology Group, Biomedical Research Institute, Hasselt University, Agoralaan Building C, Diepenbeek, Belgium; 2 Belgian Nuclear Research Centre, Radiobiology Unit, SCK•CEN, Mol, Belgium; 3 Institute of Oncology “Prof. dr. Ion Chiricuta”, Cluj-Napoca, Romania; 4 ‘Iuliu Hatieganu’ University of Medicine and Pharmacy, Department of Oral and Maxillofacial Radiology, Cluj-Napoca, Romania; 5 Paris Descartes University - Sorbonne Paris Cité, EA 2496 - Orofacial Pathologies, Imaging and Biotherapies Lab and Dental Medicine Department, Bretonneau Hospital, HUPNVS, AP-HP, Paris, France; 6 Namur Research Institute for Life Sciences, University of Namur, Namur, Belgium; 7 Katholieke Universiteit Leuven, Department of Imaging and Pathology, OMFS-IMPATH Research group, and University Hospitals, Oral and Maxillofacial Surgery, Dentomaxillofacial Imaging Center, Kapucijnenvoer 7, Leuven, Belgium; 8 Karolinska Institutet, Department Dental Medicine, Huddinge, Sweden

**Keywords:** DNA damage response, Three-dimensional imaging, Biomarkers

## Abstract

Assessing the possible biological effects of exposure to low doses of ionizing radiation (IR) is one of the prime challenges in radiation protection, especially in medical imaging. Today, radiobiological data on cone beam CT (CBCT) related biological effects are scarce. In children and adults, the induction of DNA double strand breaks (DSBs) in buccal mucosa cells and 8-oxo-7,8-dihydro-2′-deoxyguanosine (8-oxo-dG) and antioxidant capacity in saliva samples after CBCT examination were examined. No DNA DSBs induction was observed in children nor adults. In children only, an increase in 8-oxo-dG levels was observed 30 minutes after CBCT. At the same time an increase in antioxidant capacity was observed in children, whereas a decrease was observed in adults. Our data indicate that children and adults react differently to IR doses associated with CBCT. Fully understanding these differences could lead to an optimal use of CBCT in different age categories as well as improved radiation protection guidelines.

## Introduction

### Uncertainties concerning low dose ionizing radiation exposure and medical imaging

Currently, a debate exists within the radiation protection community about which model best reflects the relation between the ionizing radiation (IR) dose and the additional health risk. Several models have been described thus far. These include: the linear non-threshold (LNT) model, the linear threshold model, the hormetic model and the hypersensitivity model^1^.

Currently, the linear non-threshold (LNT) model is used to estimate risks in radiation protection guidelines. Although the LNT model is supported by epidemiological evidence in the high dose range (>100 milliGray (mGy)), increasing evidence disproves it in the low dose range^2–5^. One of the main critiques is the fact that the LNT model does not take into account biological defence mechanisms (e.g. DNA repair mechanisms)^6,7^. In addition, a lot of uncertainties still exist about low doses (<100 mGy), mostly because of a lack of statistical power of the epidemiological data. Knowing which of these models supports the relation between exposure to low doses of IR and the involved risk best is of importance in medical imaging applications of IR. Such applications include computed tomography (CT) and, more recently, cone beam computed tomography (CBCT), which typically uses doses far below 100 mGy, (typically between 0.01–0.10 mGy)^8–11^.

Multiple controversial studies indicate that exposure of children to diagnostic radiology may lead to radiation-induced malignancies later in life. Retrospective studies observed that the use of CT scans in children could triple the risk of leukaemia and brain cancers^12–14^. A 24% increase in cancer incidence was seen in an Australian linker study, which indicated exposure at younger age resulted in an increased cancer incidence^15^. The EPI-CT study was set up to gain more insight into the potential adverse effects associated with CT examinations in children^16^. Finally, it was estimated that the probability to develop radiation-induced malignancies after CBCT exposure is 6 cases per 1,000,000 CBCT scans on average, with age at exposure and gender mostly influencing the risk^17,18^. Despite these potential links between diagnostic radiology and radiation-induced malignancies, absolute evidence from prospective studies is scarce^3,8^. Yeh *et al*. (2018) estimated the risks of dental CBCT and found that the risk of exposure-induced death (REID) values were highest in 10-year old subjects. These REID values were two times higher than in 30-year old subjects. The risk was higher in females than in males. Furthermore, the risk decreased with increasing age^19^. Radiobiological research can help explain the uncertainties of epidemiological studies as well as give more insights into the underlying mechanisms^20,21^.

Since the introduction of CBCT in the late 1990s, its use has become widespread and is applied in several specialties in dental medicine including oral and maxillofacial surgery, orthodontics, periodontics and dental implants^22–24^. It is said that children are more radiosensitive than adults, therefore questions are raised about potential radiation-induced health effects associated with diagnostic radiology in children^9,10,25–28^. IR doses associated with paediatric dental CBCT became a major concern for the general public when the New York Times published two articles about the topic (2010 and 2012)^9,28–30^.

IR can cause several types of DNA lesions, including single strand breaks, double strand breaks (DSBs) and base alterations^31–33^. DNA DSBs are considered the most harmful^34^. Inaccurate repair of DSBs could result in mutations, chromosome rearrangements, chromosome aberrations and loss of genetic information^35,36^. Therefore, eukaryotes have developed the DNA damage response (DDR)^37^. The DDR consists of a signalling cascade that results in the recruitment of multiple DDR proteins to the vicinity of DSBs, including histone H2AX phosphorylated on serine 139 (γH2AX) and p53-binding protein 1 (53BP1). Both γH2AX and 53BP1 form DNA damage foci and show a quantitative relationship between the number of foci and the number of DSBs^38,39^.

Over 60% of a cell consists of water, thus most of the DNA damage caused by X-rays is indirect via free radicals such as ROS (e.g. the hydroxyl radical, superoxide radicals and hydrogen peroxide)^31,40^. An excess of reactive oxygen species (ROS), called oxidative stress, is countered by antioxidant defence mechanisms. ROS can cause oxidative DNA damage through oxidative base lesions^41–43^. An example of oxidative damage to DNA/nucleotides is 8-oxo-7,8-dihydro-2′-deoxyguanosine (8-oxo-dG). 8-oxo-dG is a base modification which is mutagenic, thus it can be sensed by DNA repair mechanisms^44^.

The buccal mucosa (BM), which lines the oral cavity, is an easily accessible source for collecting buccal mucosal cells (BMCs) in a minimally invasive, pain-free way^45^. BMCs have been used to study (amongst others) the impact of nutrition, lifestyle factors and exposure to genotoxins, including exposure to IR^46,47^. IR-induced genotoxicity can be monitored in BMCs by measuring γH2AX levels^48,49^.

Saliva is a bodily fluid that is secreted into the oral cavity. It originates mainly from the parotid, submandibular and sublingual glands and is an aqueous solution (>99% water) containing both organic and inorganic molecules^50^. Saliva, commonly referred to as ‘mirror of the body’, has several advantages over other biological samples, such as blood: It is readily available, collection can be done in a non-invasive way, and its use is very cost-effective^51,52^. Therefore saliva is an ideal sample to collect from paediatric patients^52,53^. Currently, salivary diagnostics is becoming increasingly important in radiation biomarker research^51,54^. Since X-rays induce most damage to biomolecules via ROS, measuring ROS and their effects in saliva samples could be a feasible indicator of radiation exposure.

The main aim of our study is to characterize the short-term radiation-induced effects associated with CBCT examinations, specifically in children. To this end, the sub-objectives were 1) to evaluate the induction of DNA DSBs in BMCs, and 2) to evaluate oxidative stress (by measuring 8-oxo-dG levels) as well as total antioxidant capacity in saliva samples^55^. All tests were performed both in children and adults, to identify potential age-related differences.

## Results

### Patients and dose exposure

In total, 147 children that participated in this study were 11 ± 3 years old. 73 boys and 74 girls were included. Besides, 23 adults (9 men and 14 women) that participated were 43 ± 17 years old. The average absorbed doses to the salivary glands were 1613 ± 19 µGy, 2416 ± 324 µGy and 4283 ± 353 µGy, for Promax 3D, Accuitomo 170 and NewTom VGi-evo respectively (see Supplementary Data 1)^56,57^. The study was approved by the ethical committees of the participating hospitals (see Material & Methods section).

### Power analysis

Power analysis was based on validations experiments that were performed prior to this study^55^. The number of participants (N) are the numbers described in this manuscript. The results of the power analysis indicate that the sample size was sufficient for all analyses, i.e. equal to or greater than 0.9 (Supplementary Table 1).

### DNA double strand break detection in exfoliated buccal mucosal cells before and after CBCT examination

**Figure 1:**
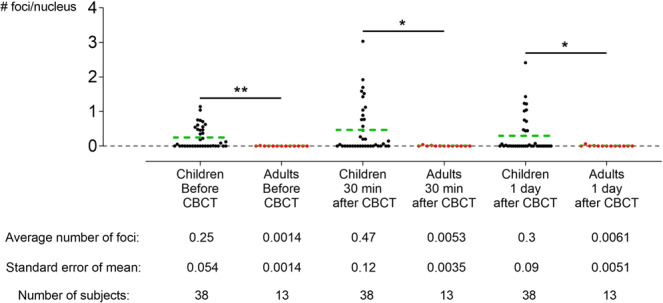
No DNA double strand breaks (DSBs) are induced in buccal mucosal cells (BMCs) after cone beam computed tomography (CBCT) examination, neither in children nor in adults. No significant increases in the amount of γH2AX/53BP1 co-localized foci were observed 30 minutes and 24 hours after CBCT examination in children (Black dots; N = 38, degrees of freedom = 2, Friedman statistic = 2.7, *p* *=* 0.2538) and in adults (Red dots; N = 13, degrees of freedom = 2, Friedman statistic = 1.0, *p* = 0.6065). Before (Mann-Whitney U value = 121, *p* *=* 0.0020), 30 minutes after (Mann-Whitney U value = 145, *p* *=* 0.0146) and 24 hours after CBCT (Mann-Whitney U value = 170, *p* *=* 0.0487) the amount of DSBs was significantly higher in children then in adults. Only the data from patients of which results were obtained for all time points were included. Green dotted line = average number of foci; *p ≤ 0.05; **p ≤ 0.0021.

The results from co-localized γH2AX and 53BP1 foci, which are a measure for DNA DSBs, show no changes in the amount of DSBs after CBCT examination, neither in children nor adults (Fig. 1).

In children (N = 38, degrees of freedom (DF) = 2, Friedman statistic = 2.7, *p* *=* 0.2538) a slight increase was seen in the amount of foci from 0.25 ± 0.054 foci/cell before CBCT to 0.47 ± 0.12 foci/cell 30 minutes after CBCT (*p* > 0.9999). 24 hours after CBCT the amount of foci returned to baseline levels (0.3 ± 0.09 foci/cell) (*p* > 0.9999). The decrease between 30 minutes after CBCT and 24 hours after, however, is not significant (*p* *=* 0.5614).

Similarly, no significant changes in the amount of co-localized γH2AX and 53BP1 foci were found in adult patients (N = 13, DF = 2, Friedman statistic = 1.0, *p* *=* 0.6065). Before CBCT, 0.0014 ± 0.0014 foci/cell were counted, which increased slightly to 0.0053 ± 0.0035 foci/cell 30 minutes after CBCT exposure (*p* > 0.9999). Contrary to the children, the number of foci per cell remained increased 24 hours after CBCT when compared to before CBCT (0.0061 ± 0.0051 foci/cell; *p* > 0.9999). Between 30 minutes after CBCT and 24 hours after CBCT no significant difference was observed (*p* > 0.9999).

Interestingly, the amount of foci per cell was significantly higher in children than in adults at every time point. Before CBCT 0.25 ± 0.054 foci/cell were observed in children and 0.0014 ± 0.0014 foci/cell were observed in adults (Before CBCT: Mann-Whitney U value = 121, *p* *=* 0.0020; 30 minutes after CBCT: Mann-Whitney U value = 145, *p* = 0.0146; and 24 hours after CBCT: Mann-Whitney U value = 170, *p* = 0.0487).

Since both children and adults showed an increase 30 minutes after CBCT, these increases were compared (# foci/cell_30 minutes after CBCT_ − # foci/cell_before CBCT_). The mean increase in children (0.17 ± 0.097 foci/cell) did not differ from the increase in adults (0.0078 ± 0.01 foci/cell) (Mann-Whitney U value = 412, *p* *=* 0.8089). Regarding the difference between 30 minutes after CBCT and 24 hours after, no significant difference was observed between children (−0.17 ± 0.11 foci/cell) and adults (0.00087 ± 0.0066 foci/cell) (Mann-Whitney U value = 196, *p* = 0.2105).

### 8-oxo-dG levels in saliva samples

**Figure 2:**
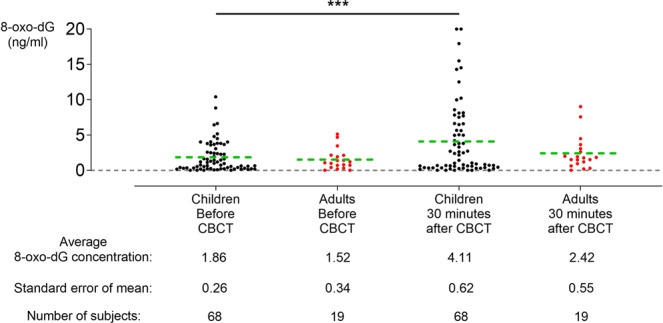
Excretion of 8-oxo-7,8-dihydro-2′-deoxyguanosine (8-oxo-dG) into saliva is increased after cone beam computed tomography (CBCT) examination in children but not in adults. Only data from patients of which results were obtained for both time points were included. In children there is a significant average increase of 121% in 8-oxo-dG excretion 30 minutes after CBCT examination (N = 68, DF = 67, t value = 4, *p* < 0.0001). In adults there is an average increase in 8-oxo-dG excretion of 59% (N = 19, DF = 18, t value = 1.58, *p* = 0.1317). Green dotted line = average; ***p < 0.0001.

8-oxo-dG levels were measured in saliva samples collected before and after CBCT examination. They were increased in children but not in adults 30 minutes after CBCT (Fig. 2).

In children, a significant increase in 8-oxo-dG levels was observed between samples taken before CBCT examination (1.86 ± 0.26 ng/ml) and 30 minutes after CBCT (4.11 ± 0.62 ng/ml) (N = 68, DF = 67, t value = 4, *p* < 0.0001), an average increase of 121% (Fig. 2). In adults, an increase from 1.52 ± 0.34 ng/ml 8-oxo-dG before CBCT to 2.42 ± 0.55 ng/ml 30 minutes after CBCT was observed (N = 19, DF = 18, t value = 1.58, *p* = 0.1317), resulting in an average increase of 59% (Fig. 2). No differences were observed between the values of children and adults before CBCT (Mann-Whitney U value = 643.5, *p* = 0.98) and 30 minutes after CBCT (Mann-Whitney U value = 622.5, *p* = 0.81).

In the group of children, data were split based on gender (Table 1). Both in boys and girls the amount of 8-oxo-dG increased significantly after CBCT examination (N = 35, *p* *=* 0.024 and N = 33, t-value = 2.91, DF = 32, *p* *=* 0.0065, respectively). Furthermore, no differences between boys and girls was observed (Table 1). This was confirmed when the proportional change between values before and after CBCT were compared between boys and girls (*p* = 0.6907) (see Supplementary Data 2).

**Table 1:** Comparison between boys and girls for 8-oxo-dG excretion before and after cone beam computed tomography (CBCT) examination.

	Boys (N = 35)	Girls (N = 33)	*P* value*	t-value	Degrees of freedom
8-oxo-dG (ng/ml)Before CBCT	1.71 ± 0.27	2.01 ± 0.46	0.63	Mann-Whitney U value = 537.5	N.A.
8-oxo-dG (ng/ml) 30 minutes after CBCT	4.21 ± 0.94	4.01 ± 0.83	0.96	Mann-Whitney U value = 573.5	N.A.
*P* value	0.024	0.0065			
t-value	(Wilcoxon test)	2.9			
Degrees of freedom	(Wilcoxon test)	32			

*For inter-group testing a paired student T-test was performed; for intra-group testing: an unpaired student T-test was performed.

**Figure 3:**
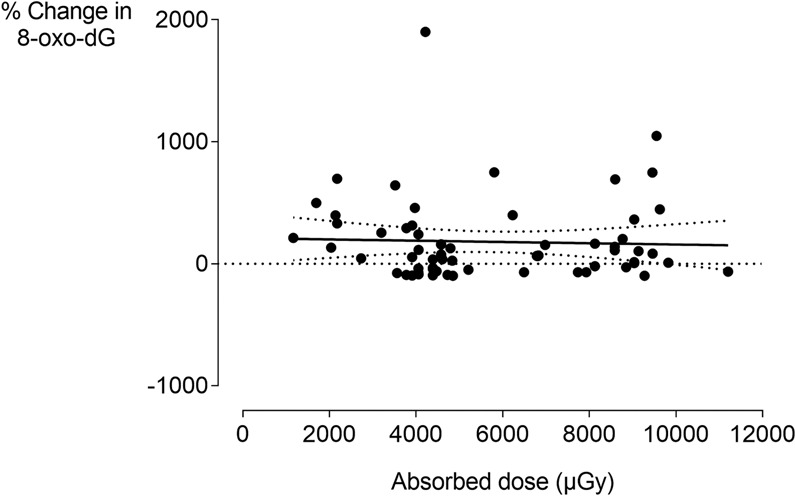
No dose response in 8-oxo-dG excretion in saliva 30 minutes after cone beam computed tomography in children. No visible dose response (linear or otherwise) was observed in 8-oxo-dG excretion in children. Radiation doses were the absorbed doses at the salivary glands as calculated by MC simulations^56,103^. Black full line: dose response curve; black dotted curved lines: 95% confidence interval of the dose response curve.

Plotting the proportional change in 8-oxo-dG levels of children against the absorbed dose received by the patients showed no visible trend or dose response (Fig. 3).

### Total antioxidant capacity in saliva samples

**Figure 4:**
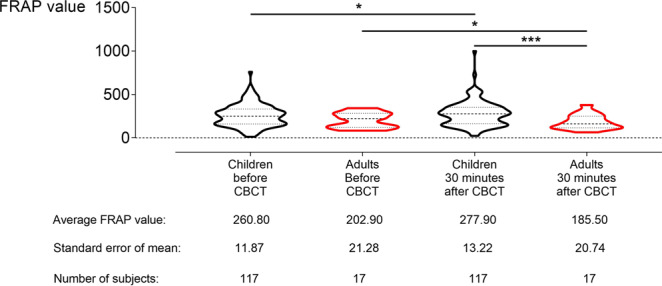
Ferric reducing antioxidant power (FRAP) values increase in saliva samples from children after cone beam computed tomography (CBCT) examination, while decreasing in saliva samples from adults. In children (black violin plots) a significant increase in FRAP values was observed 30 minutes after CBCT examination (N = 117, t-value = 1.98, degrees of freedom (DF) = 116, *p* *=* 0.0498). In adults (red violin plots) a significant decrease was observed 30 minutes after CBCT examination (N = 17, t-value = 2.22, DF = 16, *p* = 0.0412). The FRAP values 30 minutes after CBCT are significantly higher in children than in adults (Welch-corrected t-value = 3.76, DF = 30.93, *p* = 0.0007). The response in children and adults differs significantly, with an average increase of 17.10 ± 8.62 in children and an average decrease of 17.40 ± 7.84 in adults (Welch-corrected t-value = 2.96, DF = 65, *p* = 0.0043). *p ≤ 0.05; ***p ≤ 0.0002.

Ferric Reducing Antioxidant Power (FRAP) values were measured in saliva samples before and 30 minutes after CBCT examination. They were significantly increased in children and decreased significantly in adults 30 minutes after CBCT examination (Fig. 4).

Children showed a slight, but significant increase in FRAP value after CBCT examination. Thirty minutes after CBCT examination, FRAP values increased from 260.80 ± 11.87 to 277.90 ± 13.22, an increase of about 7% (N = 117, t-value = 1.98, DF = 116, *p* = 0.0498). Contrary to the results in children, a decrease of about 9% in FRAP values was found in adults. FRAP values decreased from 202.90 ± 21.28 at baseline to 185.50 ± 20.74 30 minutes after CBCT examination (N = 17, t-value = 2.22, DF = 16, *p* = 0.0412). No significant differences were observed between children and adults before CBCT examination (t-value = 1.80, DF = 132, *p* = 0.0747). However, the FRAP values 30 minutes after CBCT examination were significantly higher in children than in adults (Welch-corrected t-value = 3.76, DF = 30.93, *p* = 0.0007). The response in children and adults differed significantly when comparing the average increase in children with the average decrease in adults (Welch-corrected t-value = 2.96, DF = 65, *p* *=* 0.0043).

Results were also analysed based on gender (Table 2). In children, both boys and girls showed an increase in FRAP values, but the increase was only significant in girls (N = 62, t-value = 0.81, DF = 61, *p* *=* 0.4194 and N = 55, t-value = 2.28, DF = 54, *p* *=* 0.0268, respectively). Additionally, in both adult men and women a decrease was observed, but this was also only significant for women (N = 4, Wilcoxon test, *p* > 0.9999 and N = 13, t-value = 2.27, DF = 12, *p* = 0.0428, respectively). Furthermore, in children it was observed that the baseline levels were lower in the morning (225.10 ± 12.48) than baseline levels in the afternoon (282.30 ± 21.04) (Welch-corrected t-value = 2.34, DF = 82.42, *p* *=* 0.0217). The same was observed in adults (baseline morning: 174 ± 21; baseline afternoon: 269 ± 42), although this difference was not statistically significant (Mann-Whitney U value = 12, *p* *=* 0.0897). Therefore, the data from children were split into a morning and afternoon group. The salivary FRAP values did not significantly differ after CBCT examination if data were corrected for time of sample collection. In the morning groups, there was no significant change in both boys and girls (N = 24, Wilcoxon test, *p* *=* 0.97 and N = 10, t-value = 0.81, DF = 9, *p* = 0.7394, respectively). In the afternoon group, FRAP levels in boys did not change (N = 17, Wilcoxon test, *p* *=* 0.89). However, in girls from the afternoon group FRAP levels increased significantly (N = 24, t-value = 2.14, DF = 23, *p* *=* 0.0431).

**Table 2:** Comparison between boys and girls FRAP values before and after cone beam computed tomography (CBCT) examination.

	Boys (N = 62)	Girls (N = 55)	*P* value*	t-value	Degrees of freedom
FRAP value Before CBCT	265.90 ± 19.39	263.00 ± 16.85	0.9318	0.086	132
FRAP value 30 minutes after CBCT	277.00 ± 22.84	295.40 ± 18.35	0.4963	0.68	132
*P* value	0.4194	0.0268			
t-value	0.81	2.28			
Degrees of Freedom	61	54			

^*^For inter-group testing a paired student T-test was performed; for intra-group testing: an unpaired student T-test was performed.

## Discussion

Determining the biological effects of exposure to low doses of IR, such as those used in medical imaging, is of paramount concern in radiation protection today. This study aimed to characterize the short-term radiation-induced effects associated with CBCT examinations, specifically in children. To this end, the number of DNA DSBs was monitored in BMCs and 8-oxo-dG levels as well as total antioxidant capacity were monitored in saliva samples using previously optimized protocols^55^.

Exposure to IR can result in DSBs, which are considered very harmful, since inaccurate repair could result in mutations, chromosome rearrangements, chromosome aberrations and loss of genetic information^31,32,35,36^. Our results indicate that exposure to radiation doses used in CBCT examinations (0.184 mGy –9.008 mGy in this study) does not induce DNA DSBs in BMCs from children and adults, as observed using a microscopic γH2AX/53BP1 co-localization assay. Previously, both the γH2AX assay and the γH2AX/53BP1 assay were used to detect DNA DSBs after exposure to radiation doses used in diagnostic and interventional radiology, such as CT scans^58–60^. These studies report a significant increase in γH2AX foci in lymphocytes 1 hour after CT examination, which uses higher radiation doses than CBCT. Furthermore, our group recently showed that low doses associated with CBCT examinations are capable of inducing DNA DSBs *in vitro* in dental stem cells^61^. BMCs have also been used successfully as a biomarker for genotoxic effects, including using the γH2AX assay to detect radiation-induced DNA DSBs^48,62,63^. These studies report increase of genotoxic effects in BMCs after low dose IR exposure. Gonzalez *et al*. (2010) showed that *in vitro* exposure of BMCs to IR induces γH2AX foci^48^. Our findings are in line with previous publications focusing on genotoxicity induced by radiological examinations. In these studies, no genotoxic effects, i.e. micronucleated cells, were observed after low doses of IR, such as panoramic dental radiology and CBCT. These studies, however, all reported increases in other nuclear alterations (e.g. pyknosis, karyorrhexis and karyolysis) that are associated with increased cytotoxicity^63–66^. Recently, Preethi *et al*. (2016) reported significant increases in the number of micronucleated cells in BMCs after dental radiography in paediatric patients^62^. Furthermore, Yoon *et al*. (2009) reported a significant increase in γH2AX foci in BMCs of adults after dental radiography^67^.

Our data show 0.0014 ± 0.0014 co-localized γH2AX/53BP1 foci per cell in BMCs from adults at baseline. This number is remarkably lower than the 0.08 ± 0.02 γH2AX foci per cell in non-irradiated BMCs reported previously by Gonzalez *et al*. (2010)^48^. These different observations can be explained by the higher sensitivity of the γH2AX/53BP1 co-staining, which eliminates the detection of γH2AX foci observed during S-phase replication fork stalling^68^. In addition, Gonzalez *et al*. (2010) treated the BMCs differently, e.g. after collection they incubated the BMCs in cell growth medium at 37° Celsius, which can also affect the number of foci counted^48^.

Interestingly, we found that before CBCT examination, but also 30 minutes and 24 hours after CBCT examination, the average number of γH2AX/53BP1 foci per cell was higher in children than in adults. This observation contradicts what has been published before, namely that aging is associated with accumulation of DNA damage^69,70^. One would expect the level of DNA damage, at least before CBCT examination, to be higher in adults than in children. However, BMCs are the first barrier in the inhalation and ingestion routes. Therefore, they are exposed to several genotoxins. These can be found in environmental and lifestyle factors such as diet, mouthwash, smoke, air pollution, etc.^71–73^. These factors can, at least partially, explain our observation, since children are more sensitive to these type of genotoxins compared to adults due to age-related differences in absorption, metabolism, development and body functions^72^.

Finally, we observed that the response after CBCT examination in children did not differ significantly from that of adults. This indicates that BMCs from children after CBCT examination do not show an increased radiosensitivity compared to BMCs from adults^25–27^. These findings are in line with results from Ribeiro *et al*. (2008). They compared the genotoxic and cytotoxic effects of dental radiography between children and adults and found no significant differences in micronucleus frequency or cytotoxicity^74^. However, the radiation doses used in radiography are lower than those used in CBCT, thus this should be interpreted with caution.

This study shows that 8-oxo-dG levels excreted in saliva increased in children but not in adults 30 minutes after CBCT. Because of its mutagenic potential, excretion of 8-oxo-dG depends on cellular DNA repair mechanisms, such as nucleotide excision repair, nucleotide incision repair and Nudix hydrolase activity^75^. Therefore, a reduced DNA repair capacity may result in accumulation of 8-oxo-dG in the cells, thus resulting in a decrease in 8-oxo-dG excretion. Since DNA repair capacity was shown to decrease with age, this could explain why the concentration of 8-oxo-dG in saliva samples of adults was not increased significantly after CBCT examination, as it was in children^76,77^. Despite the significant increase in children and the limited increase in adults, no statistical differences were observed between both groups. This is most likely due to the limited group size of the adult group.

Previously, an association between the excretion of 8-oxo-dG and high radiation doses was described^78^. This association was not linear and showed saturation between 0.5 and 1 Gy. However, such dependency was not observed in this study, for example children that were exposed to 0.8 mGy showed a similar increase in 8-oxo-dG excretion as children exposed to 0.2 mGy. These data indicate that there is a high variability in individual radiosensitivity in our study population. Alternatively, it could be that the very low IR doses associated with CBCT elicit a small biological response which is unrelated to the IR dose, like an all-or-nothing mechanism. This is similar to the use of a ‘priming dose’ in adaptive response studies. Here a very low dose of a stressor (e.g. a chemical or IR) results in a small response which in turn prepares cells to an exposure of the same stressor at a higher dose^79^. Our results mimic the effects seen when applying such a ‘priming dose’.

Although 8-oxo-dG was proposed as a marker for radiosensitivity, evidence is lacking or comes from radiotherapy patients, who receive doses that are a lot higher than the doses in our study population^80^.

We describe for the first time that salivary 8-oxo-dG levels are significantly increased in both boys and girls after CBCT examination. No significant gender differences in salivary 8-oxo-dG levels were observed. Previous measurements in urine and other cells showed similar results^81–83^. To the best of our knowledge, similar findings of 8-oxo-dG secretion in saliva in children were not reported before. Previous studies analysed oxidative stress markers in adults. These studies reported higher ROS production and oxidative stress biomarkers in men when compared to premenopausal women (reviewed by Kander *et.al*.^84^). It is noteworthy that these studies are all related to cardiovascular diseases and not radiation exposure. However, there are studies that report higher oxidative status in females which contradicts the aforementioned studies^85^.

Finally, the authors want to state that these results should be interpreted cautiously, since multiple sources of 8-oxo-dG exist. Guanosine bases in the nucleotide pool can also be oxidized and detected in saliva^75,78,86,87^. Therefore, the 8-oxo-dG that was detected can also originate from the nucleotide pool, rather than from the DNA.

FRAP values give information about the total antioxidant capacity of biological samples. Our data shows on opposite response between children and adults 30 minutes after CBCT examination: salivary FRAP values increase significantly in children, whilst they decrease significantly in adults. Furthermore, the response in children is significantly different from that in adults, indicating that children react differently to CBCT-associated radiation exposure. Interpretation of the data needs to be done cautiously, since the data show that the time of sampling (in the morning or in the afternoon) significantly affected the baseline salivary FRAP values in children. The highest values were measured in the afternoon. Similar circadian changes in FRAP values were observed before^88^. After correcting for time of sampling, no significant changes in salivary FRAP levels were observed, except for girls that were sampled in the afternoon. However, since pair-wise tests were used, this circadian influence is expected to be limited in this study.

Total antioxidant capacity has been used previously as a salivary biomarker related to periodontal disease and dental caries. Decreases in total antioxidant capacity have been linked to periodontal disease^89^.

The use of total antioxidant capacity as a biomarker has several limitations. Firstly, the total antioxidant capacity that is measured is the result of a complex mixture of antioxidants that is present in saliva. The major antioxidant in saliva has been reported to be uric acid, which accounts for more than 85% of the salivary antioxidant capacity. In addition, a wide array of other potent antioxidants are found in saliva, such as superoxide dismutase, catalase, glutathione peroxidase, ascorbic acid, several vitamins and albumin^90,91^. In this regard, future analysis into the enzymatic activity of specific antioxidant enzymes, e.g. superoxide dismutase might be interesting. Secondly, a lot of biological variability of salivary total antioxidant capacity exists. We report an average salivary FRAP value of 202.90 ± 21.28 in adults at baseline, whereas an average of 610.83 ± 4.52 was reported before in healthy adults^92^. It is noteworthy that this patient population was Asian, where ours is European, which may suggest ethnical differences in salivary FRAP values. Finally, several confounding factors have been described that affect the saliva composition and can thus affect the total antioxidant capacity. Confounding factors may include circadian rhythm, gender, age and diet^88,90^. This study also found an effect of circadian rhythm (see above), age and gender. Girls show a significant increase in salivary FRAP values, whereas women show a significant decrease. Both boys and men showed a change (an increase and decrease, respectively), but this was not significant. These findings indicate that females are more susceptible to changes in total antioxidant capacity following IR exposure and that the net effects depends on the age of the individual. However, it is important to note that our patient group is relatively small (N = 72 for girls and N = 13 for women). Increasing the sample size could therefore yield different results. These limitations could interfere with interpretation of the results. Therefore, it is important to take these confounding factors into account during the design of a study. As with 8-oxo-dG, no dose response relationship was observed for FRAP values.

In conclusion, our data provide evidence that CBCT examinations causes changes in the oxidation response in children. In adults, a slight increase in 8-oxo-dG levels and a significant decrease in the antioxidant response were observed. Despite this increase in oxidation response, no induction of DNA DSBs in BMCs was observed in children nor in adults.

Since no DNA DSBs are observed, the changes in 8-oxo-dG and FRAP levels can also be explained by an adaptive response^4,93–95^. Since 8-oxo-dG excretion and antioxidant capacity both increase in children, it could indicate that the intracellular defence mechanisms are being ‘primed’, i.e. they are being prepared for a subsequent exposure to IR. Indeed, since no DNA DSBs are observed, the DNA repair mechanisms are working properly, if DNA damage was ever induced at all, and the increase in 8-oxo-dG excretion and antioxidant capacity could indicate an increase in the antioxidant defence system. However, these results are not observed in adults. Therefore, additional research into the oxidation response following CBCT examinations is required. Besides age-related differences, we observed some gender-related differences. Girls/women showed a significant increase/decrease in FRAP values after CBCT examination, whereas boys/men do not. Our data also demonstrate that saliva can be used for biomonitoring after IR exposure even if the radiation doses are very low (<1 mGy). However, no dose response relationship was found, neither for 8-oxo-dG levels nor for FRAP values.

Nonetheless, these results should raise awareness about radiation protection and the ‘As-Low-as- Diagnostically Acceptable being indication-oriented and patient-specific’ (ALADAIP) principle among clinicians and radiologists^9^. However, this should be investigated into more depth to gather more information about the potential link between possible biological effects and the CBCT settings that were used. Furthermore, the effects observed and described in this study are short-term effects, i.e. within 30 minutes after CBCT examination. We can conclude that molecular effects, although very small, occur and that further research is warranted. These findings are an incentive for continuing research into the short- and long-term biological effects after CBCT examination, especially the antioxidant response, since fully understanding them could lead to an optimal use of CBCT in a paediatric population as well as improved radiation protection guidelines.

## Materials and Methods

### EU OPERRA - DIMITRA study

The DIMITRA study is an non-interventional, prospective study that focusses on radiation-induced effects related to diagnostic CBCT exposure in children. It is a multicentre study carried out in three European centres: the Oral and MaxilloFacial Surgery – Imaging & Pathology department (Katholieke Universiteit Leuven, Leuven, Belgium), the Dental Medicine Department of the Bretonneau Hospital (Paris, France) and the Iuliu Hatieganu University of Medicine and Pharmacy (Cluj-Napoca, Romania)^55^. All experiments and methods were performed in accordance with relevant guidelines and regulations. All experimental protocols were approved by a named institutional/licencing committee. Ethical approval was obtained at the participating sites (Commissie Medische Ethiek KU Leuven, B322201525196, Belgium; Comité d’Evaluation de l’Ethique des projets de Reserche Biomédicale Paris Nord, N°16-021, France; Comisia de Etica UMF Iuliu Hatieganu Cluj-Napoca, 208/21.04.2015, Romania). In case of underage children, both parents needed to consent unless one parent has explicit permission from the other parent^55^.

### Patient selection

Patients with various indications were referred to the clinic for CBCT examination. They were examined using CBCT device settings that match their individual needs. Thus the FOV, kV, mAs and resolution mode are adjusted to fit with each individual’s indication and age, in agreement with the ALADAIP principle, as described in the DIMITRA position statement by Oenning *et al*.^9^. Throughout the three participating centres, three CBCT devices were used: Accuitomo 170 (Mortia, Osaka, Japan), NewTom VGi evo (Cefla S.C., Imola, Italy) and Promax 3D (Planmeca OY, Helsinki, Finland).

Eligible patients were children/adolescents from 3 to 18 years old, as well as adults (>18 years old), with good oral hygiene. Exclusion criteria were the presence of systemic diseases, the use of antibiotics or anti-inflammatory drugs, smoking and not giving informed consent prior to enrolment.

### Informed consent

Informed consent was obtained for each participating patient. In case of underage children, informed consent from a parent and/or legal guardian was obtained prior to inclusion in the study.

### Buccal mucosal cell collection and immunocytological staining

The collection and staining method were described in detail by Belmans *et al*.^55^. Briefly, synthetic swabs were used to collect BMCs just before, 30 minutes and 24 hours after CBCT examination using a protocol modified from Thomas *et al*.^45^. Before each swabbing the patientrinsed his/her mouth twice with water. The swabs were put in Saccomanno’s fixative (50% ethanol and 2% polyethylene glycol in milliQ water) and stored at 4 °C. Next, the BMCs were centrifuged at 580 *g* for 10 minutes. Then they were washed three times in buccal buffer (BuBu) (0.01 M Tris-HCl, 0.1 M EDTA, 0.02 M NaCl, 1% FBS, pH = 7). Next the BMCs were passed through a 100 µm nylon filter (Falcon®, VWR Belgium, Leuven, Belgium). Then the BMCs were washed one last time and pelleted. The pelleted BMCs were fixed in 500 µl of 2% paraformaldehyde (PFA) (Sigma Aldrich, St-Louis, MO, USA). Afterwards, the BMCs were washed twice with 1x phosphate-buffered saline (PBS) (Gibco, Life Technologies, Ghent, Belgium). Then they were spotted on coverslips by cytocentrifugation (ThermoFisher, Waltham, MA, USA). The coverslips were placed in 4-well culture plates (Nunc, ThermoFisher, Roskilde, Denmark) so that the BMCs were facing up.

The BMCs were washed with 1x PBS before permeabilization with 0.25% Triton X-100 in 1x PBS. After another washing step, the BMCs were blocked with 1x pre-immunized goat serum (ThermoFisher, Waltham, MA, USA) in 1x TBST and 0.005 g/v% TSA blocking powder (PerkinElmer, FP1012, Zaventem, Belgium) (TNB) for 1 hour at room temperature (RT). Afterwards, the BMCs were incubated with primary mouse monoclonal anti-γH2AX antibody (Millipore 05–636, Merck, Overijse, Belgium) (1:300 in TNB) and rabbit polyclonal anti-53BP1 antibody (Novus Biologicals NB100–304, Abdindon, UK) (1:1000 in TNB). Incubation was done overnight at 4 °C on a rocking platform. After incubation, the BMCs were washed in 1x PBS. Then the BMCs were incubated for 1 hour at RT with goat anti-mouse Alexa Fluor® 488-labelled antibody (ThermoFisher, A11001, Waltham, MA, USA) (1:300 in TNB) and goat anti-rabbit Alexa Fluor® 568-labelled antibody (1:1000 in TNB) (ThermoFisher, A11011, Waltham, MA, USA). Afterwards the BMCs were washed with 1x PBS and finally the coverslips were mounted with Prolong Diamond antifade medium with 4′,6-diamidino-2-phenylindole (DAPI) (ThermoFisher, Waltham, MA, USA).

Finally, images were acquired with a Nikon Eclipse Ti fluorescence microscope using a 40x dry objective (Nikon, Tokyo, Japan). Images were analysed with open source Fiji software^96^, which analyses each nucleus based on the DAPI signal and within each nucleus the signals from Alexa Fluor® 488 and −568 represent the γH2AX and 53BP1 foci, respectively. The number of co-localized foci per nuclei were determined using the Cellblocks toolbox^97^.

### Saliva collection

The collection of saliva samples was described in detail by Belmans *et al*.^55^. In summary, saliva samples were collected right before and 30 minutes after CBCT examination using the passive drool method^98^, and sampling coincided with the BMC collection. Immediately after collection, the whole saliva was stored at −20 °C until shipment. After shipment to the lab, saliva samples were centrifuged at 10,000 *g* at 4 °C and the supernatant was stored at −80 °C until further analysis.

### 8-oxo-dG enzyme-linked immunosorbent assay

8-oxo-dG was analysed using a 8-oxo-dG enzyme-linked immunosorbent assay (ELISA). Prior to this assay, 500 µl of saliva was purified twice on a C18 solid phase extraction column (Varian, Lake Forest, CA, USA) as described by Shakeri Manesh *et al*.^99^. The 8-oxo-dG ELISA (Health Biomarkers Sweden AB, Stockholm, Sweden) was performed as described by Haghdoost *et al*.^78^. In short, 270 µl of sample/standard was added to 165 µl of primary antibody and incubated for 2 hours at 37 °C on a shaker. The ELISA plate was washed with 1x PBS and 140 µl of sample/standard was loaded per well. The plate was incubated overnight at 4 °C on a shaker. Next, the plate was washed with 1x washing solution and 140 µl of secondary antibody was added per well. After a 2 hour incubation at RT, the plate was washed with 1x washing solution. Afterwards, 140 µl of chromogenic substrate 3,3′,5,5′-tetramethylbenzidine (One-step substrate system, Dako, Glostrup Municipality, Denmark) was added and the plate was incubated for 15 minutes at RT. The colour reaction was stopped by adding 2 M sulphuric acid. Finally, the absorbance was measured at 450 nm (signal) and 570 nm (background) using a microplate reader (ClarioStar, BMG Labtech, Ortenberg, Germany). 8-oxo-dG levels were interpolated based on a standard curve (range: 0.02–10 ng 8-oxo-dG/ml).

### Total antioxidant capacity determination

The Ferric Reducing Antioxidant Power (FRAP) assay (Cell Biolabs, CA, USA) was performed on whole saliva according to the manufacturer’s instructions. Briefly, 100 µl of sample/standard and 100 µl reaction reagent were added per well of a 96-well plate. Then the plate was incubated for 10 minutes at RT on a shaker. Finally, the absorbance was measured at 560 nm using a microplate reader (ClarioStar, BMG Labtech, Ortenberg, Germany).

### Statistics

Statistical analysis was performed using GraphPad 7.02 (GraphPad Inc., CA, USA). The results of the DNA DSBs in BMCs were analysed using repeated measures one-way analysis of variance (ANOVA). 8-oxo-dG and FRAP assay results were analysed using two-tailed paired t-tests. To analyse differences between age groups and differences in radiation sensitivity, two-tailed unpaired t-tests were performed. While all tests listed above are parametric tests, non-parametric alternatives were used if conditions were not met. *P* values lower than 0.05 were considered as statistically significant. Results are shown as mean ± standard error of the mean (SEM).

### Power analysis

Power analysis was performed in R^100^. Input values for expected differences and standard deviations, values were taken from a previously published validation study^55^. The power for the t-test with significance level 0.05 was calculated with the R-package ‘pwr’^101^. For one-way ANOVA, the power was calculated with a significance level of 0.05 using the R-package wp.rmanova^102^.

## Supplementary information

Supplementary Data.
