# Author Correction: Quantification of DNA double strand breaks and oxidation response in children and adults undergoing dental CBCT scan

**DOI:** 10.1038/s41598-024-79000-2

**Published:** 2024-11-18

**Authors:** Niels Belmans, Liese Gilles, Randy Vermeesen, Piroska Virag, Mihaela Hedesiu, Benjamin Salmon, Sarah Baatout, Stéphane Lucas, Ivo Lambrichts, Reinhilde Jacobs, Marjan Moreels, DIMITRA Research Group, A. C. Oenning, C. Chaussain, C. Lefevre, M. Baciut, M. Marcu, O. Almasan, R. Roman, I. Barbur, C. Dinu, H. Rotaru, L. Hurubeanu, V. Istouan, O. Lucaciu, D. Leucuta, B. Crisan, L. Bogdan, C. Candea, S. Bran, G. Baciut, H. Bosmans, R. Bogaerts, C. Politis, A. Stratis, R. Pauwels, K. de F. Vasconcelos, L. Nicolielo, G. Zhang, E. Tijskens, M. Vranckx, A. Ockerman, E. Claerhout, E. Embrechts

**Affiliations:** 1 Morphology Group, Biomedical Research Institute, Hasselt University, Agoralaan Building C, Diepenbeek, Belgium; 2 Belgian Nuclear Research Centre, Radiobiology Unit, SCK•CEN, Mol, Belgium; 3 Institute of Oncology “Prof. Dr. Ion Chiricuta”, Cluj-Napoca, Romania; 4 ‘Iuliu Hatieganu’ University of Medicine and Pharmacy, Department of Oral and Maxillofacial Radiology, Cluj-Napoca, Romania; 5 Paris Descartes University-Sorbonne Paris Cité, EA 2496 - Orofacial Pathologies, Imaging and Biotherapies Lab and Dental Medicine Department, Bretonneau Hospital, HUPNVS, APHP, Paris, France; 6 Namur Research Institute for Life Sciences, University of Namur, Namur, Belgium; 7 Katholieke Universiteit Leuven, Department of Imaging and Pathology, OMFS-IMPATH Research group, and University Hospitals, Oral and Maxillofacial Surgery, Dentomaxillofacial Imaging Center, Kapucijnenvoer 7, Leuven, Belgium; 8 Karolinska Institutet, Department Dental Medicine, Huddinge, Sweden

Correction to: *Scientific Reports* 10.1038/s41598-020-58746-5, published online 07 February 2020

This Article contains an error in the Results section, under the subheading ‘DNA double strand break detection in exfoliated buccal mucosal cells before and after CBCT examination’.

“The mean increase in children (0.17 ± 0.097 foci/cell) did not differ from the increase in adults (0.0078 ± 0.01 foci/cell) (Mann-Whitney U value = 412, *p* = 0.8089).”

should read:

“The mean increase in children (0.22 ± 0.097 foci/cell) did not differ from the increase in adults (0.0078 ± 0.01 foci/cell) (Mann-Whitney U value = 412, *p* = 0.8089).”

