# Supplementary material for: Quantification of DNA Double Strand Breaks and Oxidation Response in Children and Adults Undergoing Dental CBCT Scan
Source: Sci Rep. 2020 Feb 7;10:2113. doi: 10.1038/s41598-020-58746-5 (PMC7005754; doi:10.1038/s41598-020-58746-5)
Supplement: Supplementary file 1 — Supplementary Data. [file 41598_2020_58746_MOESM1_ESM.pdf]

Quantification of DNA double strand breaks and oxidation response in children and adults undergoing dental CBCT scan.

Authors

Niels Belmans[1,2], Liese Gilles[1], Randy Vermeesen[2], Piroska Virag[3], Mihaela Hedesiu[4], Benjamin Salmon[5], Sarah Baatout[2], Stéphane Lucas[6], Ivo Lambrichts[1], Reinhilde Jacobs[7,8], Marjan Moreels[2*] & DIMITRA Research Group

[1] Morphology Group, Biomedical Research Institute, Hasselt University, Agoralaan Building C, Diepenbeek, Belgium

[2] Belgian Nuclear Research Centre, Radiobiology Unit, SCK•CEN, Mol, Belgium

[3] Institute of Oncology "Prof. dr. Ion Chiricuta", Cluj-Napoca, Romania

[4] 'Iuliu Hatieganu' University of Medicine and Pharmacy, Department of Oral and Maxillofacial Radiology, , Cluj-Napoca, Romania

[5] Paris Descartes University - Sorbonne Paris Cité, EA 2496 - Orofacial Pathologies, Imaging and Biotherapies Lab and Dental Medicine Department, Bretonneau Hospital, HUPNVS, AP-HP, Paris, France

[6] Namur Research Institute for Life Sciences, University of Namur, Namur, Belgium

[7] Katholieke Universiteit Leuven, Department of Imaging and Pathology, OMFS-IMPATH Research group, and University Hospitals, Oral and Maxillofacial Surgery, Dentomaxillofacial Imaging Center, Kapucijnenvoer 7, Leuven, Belgium

[8] Karolinska Institutet, Department Dental Medicine, Huddinge, Sweden

*: Corresponding author:

Dr. Marjan Moreels

Belgian Nuclear Research Centre

Institute for Environment, Health and Safety – Interdisciplinary Biosciences – Radiobiology Unit

Boeretang 200

2400 Mol

marjan.moreels@sckcen.be

+32 14 33 28 16

Supplementary Table 1

Supplementary Table 1. Power analysis for one-way repeated analysis of variance and pair t-tests.

	Statistical test	N	Effect size (F or Cohen)**	Significance level	Power
DNA damage: children	One-way RM ANOVA*	38	2.2	.05	1
DNA damage: adults	One-way RM ANOVA	13	2.2	.05	.9
8-oxo-dG: children	Paired t-test (2-sided)	68	.666	.05	.99
8-oxo-dG: adults	Paired t-test (2-sided)	19	0.833	.05	.93
FRAP: children	Paired t-test (2-sided)	117	1.888	.05	1
FRAP: adults	Paired t-test (2-sided)	17	1.888	.05	.99

*: RM ANOVA = repeated measures analysis of variance
**: F value for one-way RM ANOVA and Cohen's effect size for paired t-tests.[58,59]

Supplementary Data 1

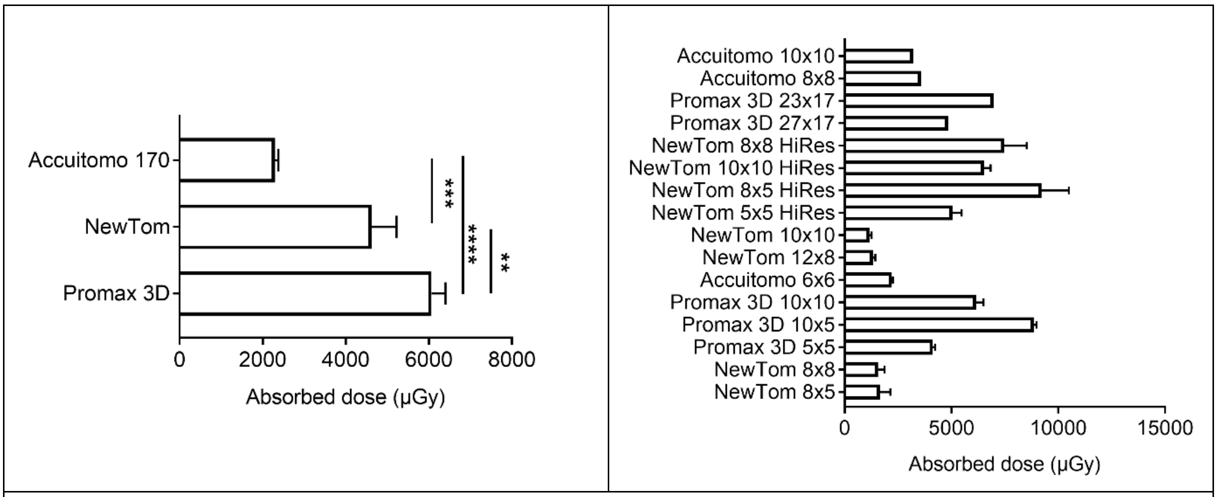

Supplementary Data 1. Absorbed radiation dose does not only depend on the device uses, but also on the field of view (FOV) and scanning protocol used during the examination. (left). Patients examined using a Promax 3D device receive on average a higher radiation dose than those examined with a Accuitomo 170 device or NewTom device. However, these data do not take into account the FOV or the scanning protocol. **(right).** Radiation dose increases with increasing FOV and resolution of the scan. This is seen for all devices (except for Accuitomo 170 for which only one scanning protocol was used). Furthermore, the radiation dose is higher when high resolution (HiRes) protocols were used. This explains the differences seen in the left panel, since for Planmeca ProMax and NewTom HiRes protocols were used, whereas only standard protocols were used in Accuitomo. No significances were shown in the right panel. ***: $p < .0002$; ****: $p < .0001$.

Supplementary Data 2

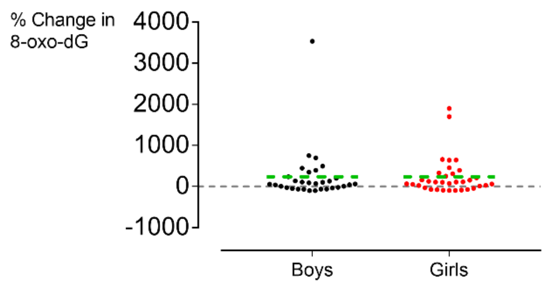

	Boys	Girls
Average % change:	239	237
Standard error of mean:	117	77

Supplementary Data 2. Comparison of % change in 8-oxo-dG excretion shows no difference between boys and girls. The proportional change in 8-oxo-dG levels does not differ between boys and girls (Mann-Whitney U value = 431, p = .203). Green dotted line = average.
